# Acknowledgement to Reviewers of *Biosensors* in 2017

**DOI:** 10.3390/bios8010005

**Published:** 2018-01-09

**Authors:** Biosensors Editorial Office

**Affiliations:** MDPI AG, St. Alban-Anlage 66, 4052 Basel, Switzerland

Peer review is an essential part in the publication process, ensuring that Biosensors maintains high quality standards for its published papers. In 2017, a total of 61 papers were published in the journal. Thanks to the cooperation of our reviewers, the median time to first decision was 19 days and the median time to publication was 47 days. The editors would like to express their sincere gratitude to the following reviewers for their time and dedication in 2017:
Abalde-Cela, SaraLiu, QingAl Ahmad, MahmoudLocatelli, MarcelloAl Machot, FadiLombardi, John R.Alander, JarmoLórenz-Fonfría, Víctor A.Ali, Md AzaharMach, PavelAnthony, CarlMadasamy, ThangamuthuAnzai, Jun-ichiMaercker, ChristianAriño Blasco, CristinaManzanera, SilvestreAubrey, DougMarquardt, DrewBachmann, MartinMartel-Foley, JosephBadea Doni, MihaelaMassaouti, MariaBaldrighi, MicheleMayer, ChristianBeni, ValerioMénard-Moyon, CéciliaBhattacharya, SayakMilano, FrancescoBirznieks, IngvarsModlitbova, PavlinaCaffarena, GabrielMondal, KunalCai, ZhongyuMoscone, DanilaCampanella, LuigiNakano, MichihikoCeylan, HakanNardelli, MimmaChamberlain, Michael DeanNg, AndyChampion, PaulNg, AlphonsusChang, JingboNi, Melody(Zhifang)Chiadò, AlessandroNikolelis, Dimitros P.Chien, Jun-ChauNiles, Andrew L.Cho, Nam-JoonOtto, CeesChoi, YongkiPace, RonChristaras, DimitriosPal, SudeshnaChun, HongguPalego, CristianoComby, StevePallaoro, AlessiaCong, LongqingPan, Tung-MingCortese, BarbaraPapanikolaou, NikolaosCosta, Maria Do Céu Gonçalves DaPappas, DimitriCostoya, Jose A.Pasluosta, CristianCrippa, PaoloPatrick, ErinCristea, CeciliaPedrero, MariaDaniele, MichaelPita, MarcosDas, JagotamoyPleissner, DanielDavis, FrankPotamitis, IlyasDe Simone, Salvatore GiovanniPu, HaihuiDi Bartolomeo, AntonioRadecka, HannaDina, Nicoleta ElenaRamadoss, AnanthakumarDoeven, EganRamer, GeorgDuncan, Thomas MRamos, DanielEgawa, YuyaRawassizadeh, RezaEl Far, OussamaRen, YongFerhan, Abdul RahimRieger, RobertFernández-Soto, PedroRinnert, EmmanuelGanesana, MallikarjunaraoRodríguez Ruiz, IsaacGauglitz, GünterRodriguez-Manzano, JesusGhafar-Zadeh, EbrahimSala, LucaGheorghiu, EugenSánchez, Beatriz JuradoGhica, Mariana EmiliaSardesai, NaimishGhosh, SohamSchneider, Ian C.Göröcs, Zoltán S.Seydou, MahamadouGrenier, KatiaShim, JiwookGunawardana, UpulShin, Dong JinHackett, MarkShityakov, SergeyHackett, Mark JSingh, Surya P.Hainberger, RainerSmith, JosephHamida, HallilSoler Aznar, MariaHe, YujieSolomon, MelaniHeitman, LauraSomnath, SuhasHirakawa, HidehikoSousa Góes, MárcioHirtz, MichaelStasiuk, GraemeHu, NingStassi, StefanoHuang, Hsi-YaStedtfeld, RobertHuang, Jin-HuaSun, Yung-ShinHuh, Yun SukSun, XiangchengHunt, Heather K.Tamassia, NicolaHuotari, MattiTarasov, AlexeyHussein, MohamedTaylor, Maureen E.Inal, SahikaTee, Benjamin C.K.Ino, KosukeTeixeira, Marcos F.S.Iyota, TaketoshiTeodorescu, FlorinaJolly, PawanThomas, SabuKeenan, JoanneTrivedi, Evan R.Kim, Jong-HoonTseng, PeterKim, Hak YongTurco, AntonioKinsella, MattVaculovičová, MarketaKirsanov, DmitryVashist, Sandeep KumarKoh, AhyeonVenkatesh, A.G.Koh, Chung-YanViefhues, MartinaKomor, AlexisVrana, Nihal EnginKong, Siu-KaiWalsh, MichaelKoo, ChiwanWang, Chiu-YenKopra, KariWang, ZheKoren, KlausWang, ShaopengKost, Gerald J.Wasisto, Hutomo SuryoKrólicka, AgnieszkaWen, Amy M.Kumeria, TusharWittenberg, NathanLakard, BorisXianyu, YunleiLakshminarayana, PolavarapuXie, Yangbo "Abel"LATOUCHE, CamilleXu, SenLedo, AnaYamada, HiroyukiLee, Jung-JaeYAO, QiaofengLee, Chee HueiYim, ChangyongLee, Boon GiinYoon, HyeonseokLeeper, FinianYuan, MingquanLei, ChongZebelo, Simon AtsbahaLemon, ChristopherZeiri, LeilaLi, HaoZeng, ShuwenLi, PengZhang, QijinLi, HeZhang, JingjingLiu, ChuanjunZhao, ZhanLiu, JingZhou, Xiao DongLiu, Weiting


